# Phleboviruses detection in *Phlebotomus perniciosus* from a human leishmaniasis focus in South-West Madrid region, Spain

**DOI:** 10.1186/s13071-016-1488-3

**Published:** 2016-04-13

**Authors:** Maria Elena Remoli, Maribel Jiménez, Claudia Fortuna, Eleonora Benedetti, Antonella Marchi, Domenico Genovese, Marina Gramiccia, Ricardo Molina, Maria Grazia Ciufolini

**Affiliations:** National Reference Laboratory for Arboviruses, Department of Infectious, Parasitic and Immune-Mediated Diseases, Istituto Superiore di Sanità, 00161 Rome, Italy; Unidad de EntomologíaMédica, Servicio de Parasitología, Centro Nacional de Microbiología, Instituto de Salud Carlos III, Ctra. Majadahonda-Pozuelo s/n, 28220 Majadahonda, Madrid Spain; Unit of Global Health, Department of Therapeutic Research and Medicines Evaluation, Istituto Superiore di Sanità, 00161 Rome, Italy; Unit of Vector-borne Diseases and International Health, Department of Infectious, Parasitic and Immune-Mediated Diseases, Istituto Superiore di Sanità, 00161 Rome, Italy

**Keywords:** *Phlebovirus*, *Phlebotomus perniciosus*, Toscana virus, Human leishmaniasis, Arbia virus, Novel virus, Madrid, Spain

## Abstract

**Background:**

*Phlebotomus*-borne (*Ph*B-) viruses are distributed in large areas of the Old World and are widespread throughout the Mediterranean basin, where recent investigations have indicated that virus diversity is higher than initially suspected. Some of these viruses are causes of meningitis, encephalitis and febrile illnesses. In order to monitor the viral presence and the infection rate of *Ph*B-viruses in a recently identified and well characterized human zoonotic leishmaniasis focus in southwestern Madrid, Spain, a sand fly collection was carried out.

**Methods:**

Sand fly insects were collected in four stations using CDC light traps during 2012–2013 summer seasons. Screening for *Phlebovirus* presence both *via* isolation on Vero cells and *via* polymerase chain reaction (PCR), using degenerated primers targeting a portion of the L segment, was performed. The serological identity and phylogenetic relationships on the three genomic segments of the viral isolates were carried out.

**Results:**

Six viral isolates belonging to different serological complexes of the genus *Phlebovirus* were obtained from fifty pools on a total of 963 *P. perniciosus* (202 females). Phylogenetic analysis and serological assays allowed the identification of two isolates of Toscana virus (TOSV) B genotype, three isolates strongly related to Italian Arbia virus (ARBV), and one isolate of a novel putative *Phlebovirus* related to the recently characterized Arrabida virus in South Portugal, tentatively named Arrabida-like virus. Positive male sand fly pools suggested that transovarial or venereal transmission could occur under natural conditions.

**Conclusions:**

Our findings highlighted the presence of different *Phlebovirus* species in the South-West area of the Madrid Autonomous Community where an outbreak of cutaneous and visceral human leishmaniasis has been recently described. The evidence of viral species never identified before in Spain, as ARBV and Arrabida-like virus, and TOSV B genotype focus stability was demonstrated. Environmental aspects such as climate change, growing urbanization, socio-economic development could have contributed to the genesis of this wide ecological niche of *Ph*B-viruses and *Leishmania * spp. The potential role of vertebrates as reservoir for the phleboviruses identified and the possibility of Phleboviruses-*Leishmania* co-infection in the same sand fly should be assessed. Furthermore the *Ph*B-viruses impact on human health should be implemented.

## Background

Phlebotomine sand flies are documented vectors of human disease agents including parasitic protozoa (*Leishmania* spp.), bacteria (*Bartonella bacilliformis*) and viruses. *Phlebotomus* borne (*Ph*B-) viruses belong to the genus *Phlebovirus* (family Bunyaviridae), *Vesiculovirus* (family Rhabdoviridae) and *Orbivirus* (family Reoviridae) are distributed in large areas of the Old World (southern Europe, Africa, the Middle East, central and western Asia) and are widespread throughout the Mediterranean region [1–3]. Some of the viruses belonging to the genus *Phlebovirus* are relevant as an emerging human health problem. Among them, Sand fly Fever Sicilian Virus (SFSV) and Sand fly Fever Naples Virus (SFNV) are the causative agents of transient febrile illness in humans, while Toscana virus (TOSV) exhibits peculiar neurotropism. Indeed, TOSV infection has been associated with aseptic meningitis or, less frequently, meningoencephalitis or encephalitis without meningitis [4–6]. Asymptomatic or mild infections were also reported in countries where TOSV circulates [7]. Due to their three segmented genome organization [Large (L), Medium (M) and Small (S), segments that encode the RNA-dependent RNA polymerase (L), envelope glycoproteins (G1 and G2), and nucleocapsid (N) with non-structural (NS) proteins, respectively] [8], genetic molecular evolution by antigenic drift, antigenic shift (genetic reassortment), and genetic recombination could be recurrent [9, 10] allowing the appearance of new variants. Indeed, recent investigations have indicated that *Phlebovirus* diversity in the Mediterranean basin is higher than initially suspected and novel viruses are yearly discovered [1, 11, 12].

Epidemiological studies in Spain have demonstrated, up to now, the circulation of two different *Ph*B-viruses belonging to the genus *Phlebovirus*: TOSV and Granada virus (GRV). The first human case of TOSV infection was revealed, by serological investigation (1986–1989), in a Swedish tourist returning from Catalonia (north-east of Spain) [13]. The first TOSV infections involving the central nervous system were reported in 1988 in Granada, where isolation of TOSV was obtained also from sand flies [14, 15]. In subsequent years, cases of TOSV infections were detected in other areas of Spain such as Murcia [16], Majorca [17], Catalonia and Madrid [18]. GRV, closely related to Massilia virus (MASV), was detected for the first time in 11 pools of *Phlebotomus * spp. in Granada province (southeastern Spain) during 2003, and its presence in phlebotomine sand flies was reported in subsequent years also in other areas of Spain (Balearic Islands and Catalonia) [19]. Anti-GRV IgM antibodies analysis carried out in acute-phase serum of individuals living in Granada province showed that GRV may cause mild infections in humans, usually a self-limited febrile illness accompanied by other signs and symptoms [20].

The Madrid area (central Spain), investigated in the present study, has been recently characterized as a relevant focus of human zoonotic leishmaniasis. In this area, leishmaniasis is endemic in rural, periurban and suburban areas and from July 2009 to date, a cutaneous and visceral leishmaniasis outbreak by *Leishmania infantum* is ongoing resulting up to now in the largest reported community outbreak of leishmaniasis in Europe [21]. The majority of cases come from the municipality of Fuenlabrada, with an incidence of 44.55 cases per 100,000 inhabitants (data provided by Boletín Epidemiológico de la Comunidad de Madrid-http://www.madrid.org/cs/Satellite?boletin=Si&c=CM_Publicaciones_FA&cid=1142685874033&language=es&pageid=1265797458663&pagename=PortalSalud%2FCM_Publicaciones_FA%2FPTSA_publicacionServicios&site=PortalSalud&volver=Si). The epidemiological link between phleboviral and *Leishmania* human infections has been widely highlighted in different European countries, both in phlebotomine and invertebrates acting as possible vectors and reservoirs of these pathogens [3, 22–26]. Only two sand fly species, *Phlebotomus perniciosus* and *Phlebotomus ariasi,* out of the seven *Phlebotomus* spp. described in the Madrid region, are proven vectors of *L. infantum* with *P. perniciosus* being the main vector [27, 28]. *P. perniciosus* was also a proven *L. infantum* vector in Fuenlabrada municipality [29, 30].

Due to the closed relationship between *Ph*B-viruses, leishmaniasis and the geographical distribution of the sand fly vectors, virological, parasitological and entomological surveillance activity represents an important tool for the control of the spread of these pathogens. In the framework of the European FP7 EDENext project for the surveillance of phlebotomines and fly-borne diseases, the objective of this work was to monitor the *Ph*B-viruses presence and infection rate in the recent human zoonotic leishmaniasis focus of the South-West area of Madrid, Spain [21].

## Methods

### Trapping stations and sand flies collection

**Fig. 1:**
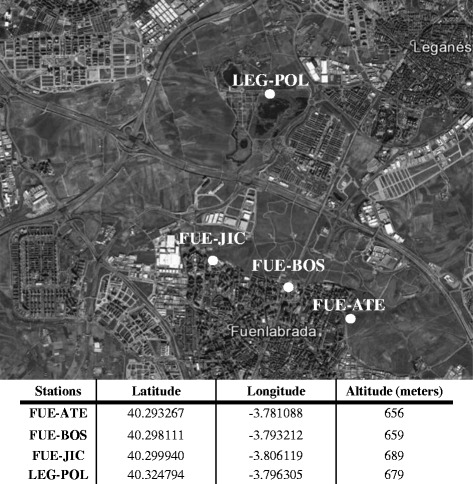
Map showing the sand fly capture stations in a periurban area of the South-West of Madrid

The sampling of sand flies was performed in a newly constructed periurban green park of around 450 ha, surrounded by large urban densely populated areas of southwestern Madrid region. Afforestation of this park began in 2005, finishing in 2011 [31]. The previous entomological surveys showed that *P. perniciosus* is the prevalent sand fly in the area at high densities (152.6 sand flies/m^2^) [29, 30, 32]. Sampling of sand flies for the present study was carried out during two following vector activity seasons (July–September 2012 and 2013) by using CDC light traps, the most suitable collection method for *Phlebovirus* isolation [33], in four peridomestic stations, named FUE-JIC, FUE-BOS, FUE-ATE (all three located in the municipality of Fuenlabrada) and LEG-POL (located in the municipality of Leganés) (Fig. 1). The selected sites were restricted to an area that had been surveyed during four consecutive years (2012–2015) and found monospecific for *P. perniciosus*. Only few specimens of a different sand fly genus, *Sergentomyia minuta,* were previously identified. This was a technical advantage for the present study because it required the immobilization of sandflies with CO_2_, specimens were then placed in a glass Petri dish over a portable chill table for the separation of both species under a stereomicroscope. Erect or recumbent abdominal setae on the posterior margins of abdominal tergites 2–6 were used for the fast identification of the genera *Phlebotomus* and *Sergentomyia*, respectively. Secondary aspects such as the general shape of the body and its color were also helpful in the identification process.

Pools of sand flies for the analysis of *Ph*B-viruses were subsamples of total catches obtained during seasonal dynamics studies carried out in the same area. Sand fly monospecificity was confirmed when the taxonomic identification of the remaining specimens collected at the same stations and dates of 2012–2013 was performed. Thus, *P. perniciosus* was the only species of the genus *Phlebotomus* identified (95.3 %) while *S. minuta* was found in a very low proportion (4.7 %) [32]. Standard morphological procedures which required female clarification and permanent mounting of specimens (males and females) in Hoyer medium and taxonomic keys [34, 35] were used.

Collected sand flies for virus isolation were pooled of ~20 specimens separated by species and sex and immediately stored at −80 °C until examination.

### Sand fly processing

All sand fly pools were homogenized, suspended in 1 mL of Hank’s solution containing 7.5 % bovine albumin and 1 % antibiotic-antimycotic mix (Invitrogen, Gibco) and centrifuged at 3,000 × g for 30 min [36]. One hundred μL of the supernatants were immediately inoculated in Vero cells to perform viral isolation and 140 μL were used for molecular identification. The remaining supernatant was stored in two aliquots at −80 °C until processing.

### Phleboviruses RNA screening by generic nested Retro Transcriptase (RT)-PCR

The RNA was extracted from supernatant aliquots by using the QIAamp viral RNA kit (Qiagen Inc., Valencia, CA, USA) according to the manufacturer’s protocol. *Phlebovirus* detection was performed amplifying *Phlebovirus* RNA-dependent RNA polymerase gene on a portion of L segment using degenerated primers [37]. The RT- and nested-PCR were performed using Super script One step RT-PCR System Kit (Invitrogen, Gaithersburg, MD) and PCR SuperMix (Invitrogen) respectively, according to the manufacture’s recommendations. The PCR conditions were those previously described [37]. PCR products were analyzed in a 2 % TAE agarose electrophoresis gel.

### Virus isolation

The virus isolation was carried out as described by Verani et al. [38]. Briefly, 100 μL of the supernatant fluid was inoculated in confluent Vero cells monolayer cultured in Nunc™ Cell Culture Tubes (Thermo Fisher Scientific Inc.*,* Waltham, MA, USA). After 1 h incubation at 37 °C in an atmosphere containing 5 % CO_2_, 2 mL of medium, consisting of Dulbecco’s MEM (DMEM), 2 % FBS and 1 % antibiotic-antimycotic mix (Invitrogen, Gibco), was added. The tubes were incubated at 37 °C and observed daily up to 14th day after inoculation to monitor the cytopathic effect (CPE). The culture samples showing the CPE were frozen at −80 °C. Viral stocks of the isolates were obtained by propagation on Vero cells and then stored at −80 °C in aliquots until use. The titration of virus stocks was carried out on six-well plates containing confluent monolayers of Vero cells infected with serial 10-fold dilutions of viral supernatants. Cells were incubated at 37 °C for 6–14 days under an overlay consisting of DMEM, 2 % FBS, 1 % antibiotic-antimycotic mix (Invitrogen, Gibco) and 2 % tragacant gum (Sigma Aldrich). The plaques were counted after staining with a solution of crystal violet (0.2 % in 10 % formaldehyde and 20 % ethanol) and the viral titre was expressed as Plaque Forming Units (PFU)/mL. The re-isolation on Vero cells of each isolate was carried out.

The culture samples showing no CPE up to the 14th day were stored at −80 °C and then used for blind passages on Vero cells. At least three passages on Vero cells were performed.

The overall virus isolation was assessed by calculating the Minimum Field Infection Rate (MFIR)/100 sand fly specimens. This value is obtained by the ratio of the number of positive pools to the total number of tested sand flies in the sample assuming that only one infected specimen is present in a positive pool [38].

### Serological identification by Plaque Reduction Neutralization (PRN) tests

PRN assays were performed, as previously described [39], using six homemade mouse immune ascitic fluids (MIAF) against SFNV, TOSV, ARBV, Teheran virus (TEHV), SFSV, and SALV. Briefly, inactivated MIAFs were diluted in two-fold dilutions starting from 1:10 and mixed with an equal volume containing 80 PFU of virus at a final volume of 100 μL. The mixture was incubated overnight at +4 °C and, the day after, inoculated into Vero cells monolayers. After adsorption, the plates were overlaid with 2 % gum tragacanth in DMEM, incubated 7–14 days at 37 °C in a humidified atmosphere containing 5 % CO_2_ and stained with a crystal violet solution. A reduction of 50 and 80 % in number of plaques was selected as criterion for virus neutralizing titres. PRN test titres < 1:10 for serum were considered negative.

### Phylogenetic analysis on L, M and S partial sequences

RNAs were extracted from isolates viral stocks by using the QIAamp viral RNA kit and RT-PCR amplifications of portions of L, M and S segments were performed. A partial coding region of L sequences of all isolates were obtained by amplification with the same degenerated primers used as screening in this study to detect the presence of *Phlebovirus* RNA [37]. The primers targeting a partial coding region of M segment and amplifying the glycoprotein G2 were designed by alignment of several Phleboviruses belonging to SFNV complex (TOSV H/IMTSSA Acc No. FJ153284; TOSV EsPhGR40 Acc No. FJ153283, TOSV Phl 32-1981-FI Acc No. DQ479914, MASV W Acc No. EU725772, GRV GR25 Acc No. GU135607, TEHV I-47 Acc No. JF939847, SFNV Acc No. HM566171, Arrabida virus PoSFPhlebV/126/2008 Acc No. KF286394) and Salehabad (SALV) complex (SALV I-81 Acc. No JX472404, ARBV PHL18 Acc. No JX472401). The degenerated primer pairs used were: Phl_M_for: GGN TAY GGN TGY TTY AAY GTN and Phl_M_rev: NCC YTC RTC RCA NGA RTA RCA; they were used at 0.4 μM per reaction in a cycling program of the RT-PCR of 50 °C for 30 min and 94 °C for 2 min, followed by 40 cycles at 94 °C for 30 s, the annealing temperature for 1 min at 52 °C, and 68 °C for 45 s, with a final elongation step at 68 °C for 7 min. To amplify a part of S coding region, two different primer pairs were used: (i) conventional degenerated consensus primers specific for SFNV complex of Phleboviruses [40] and (ii) degenerated primers specific for SAL complex of Phleboviruses designed by sequence alignments of ADANA virus (Acc No. KJ939332), SALV (Acc No. JX472405) and ARBV sequence (Acc No. JX472402). The primer pairs used were: Phl_S_Sal_for: CCARGGWTATGAYGCTGCTMG and Phl_S_Sal_rev: GGCTGYTCAAARCTCTTKRSWAC; they were used at 0.4 μM per reaction. The cycling program of the RT-PCR consisted of 50 °C for 30 min and 94 °C for 2 min, followed by 40 cycles at 94 °C for 30 s, the annealing temperature for 1 min at 55 °C, and 68 °C for 1 min, with a final elongation step at 68 °C for 7 min.

The amplicons of partial L, M and S segments were sequenced and the sequences of 438 bp, 449 bp and 258 bp respectively, were aligned with other *Phlebovirus* sequences present in GenBank using the program ClustalW (www.clustal.org/) as implemented in the Bio-Edit software version 7.2.5 [41]. DAMBE software version 5.5.29 was utilized for the adjustment of the nucleotide alignment in respect to the correlate amino acid alignment. Maximum Likelihood Model Test as implemented in Mega version 6.06 software was utilized for each segment alignment to carry out statistical selection of best-fit models of nucleotide substitution. For L and M segment the best fitting model was T92 + G; instead K2 + G + I appeared the best fitting model for S segment.

For each of three partial segments, phylogenetic trees were constructed with MEGA software utilising the parameters of analysis indicated by ModelTest with 1,000 bootstrap reiterations. The same procedures were utilised also for the amino acid translation of each segment. Distances among sequences for each gene segment were calculated with MEGA software without any correction for different evolutionary models to better evaluate the absolute distances among sequences.

The sequences of the isolates had been submitted to GenBank, and the corresponding accession numbers are given in Table 2.

## Results

### Sand fly trapping and *Phlebovirus* detection

The selected collection sites have been restricted to an area where *P. perniciosus* resulted to be the only species of the genus *Phlebotomus* collected at high densities (152.6 sand flies/m^2^) [29, 30, 32] and therefore the only vector of *L. infantum* present. Mapping of sand fly collection stations, close to an urban park, is reported in Fig. 1. A total of 963 *P. perniciosus* specimens were processed and tested for *Ph*B-viruses presence, 366 (173 females) for 20 pools in 2012 and 597 sand flies (29 females) for 30 pools in 2013 (Table 1). The sex ratio was 1.1/1.0 (52.7 % males and 47.3 % females) in 2012 and 19.0/1.0 (95.3 % males and 4.7 % females) in 2013.

**Table 1:** Collection sites, and *Ph*B-virus isolates in *Phlebotomus perniciosus* captured in Fuenlabrada and Leganés Municipalities

Collection site code	Date	2012			2013		
		M	F	M + F	M	F	M + F
		No. of sand flies/No. of pools			No. of sand flies/No. of pools		
FUE-ATE	July	20/1	16/1		53/2	–	
	August	20/1	17/1		27/1	–	
	September	–	–	22 (11 F)/1	40/2	–	
FUE-BOS	July	15/1	24/1		71/3	19/1	
	August	21/2 [1]	17/1		43/2 [1]	–	
	September	–	–	13 (1 F)/1	38/2	–	
FUE-JIC	July	21/1	24/1 [1]	15 (11 F)/1	103/5 [1]	9/1	
	August	23/1 [1]	31/1		74/4	–	
	September	–	–	12 (2 F)/1	60/3	–	
LEG-POL	July	–	–		–	–	
	August	26/1 [1]	11/1				19 (1 F)/1
	September	–	–	18 (8 F)/1	41/3	–	
Total		146/8 [3]	140/7 [1]	80 (33 F)/5 [0]	550/27 [2]	28/2 [0]	19 (1 F)/1 [0]
Total 2012–2013		366/20 [4]			597/30 [2]		

[] positive pools, FUE Fuenlabrada, LEG Leganés, M male, F female

The results obtained by generic RT-PCR plus nested-PCR amplifying a portion of the L segment and showed positivity in 4 pools of sand flies collected in 2012 (Fue-Sp40, Fue-Sp42, Fue-Sp45, Leg-Sp49) and in 2 pools of sand flies collected in 2013 (Fue-Sp136 and Fue-Sp149) (Tables 1 and 2). All 6 pools showed CPE after inoculation on Vero cell cultures. The incubation period for the production of plaques on Vero cells was 7 days for the isolates Fue-Sp40-42-45-136 and Leg-Sp49 and 14 days for isolate Fue-Sp149; the plaques morphology was different for the different isolates. No CPE was observed from cell cultures inoculated with the other 44 pools. The amplicons of partial L, M and S genomic segments obtained by RT-PCRs from all six isolates using degenerated primers were sequenced. The blast of L, M and S sequences, [EMBL: LN848240–256] with other *Phlebovirus* sequences, available in the GenBank, identified: the isolates Fue-Sp45 and Fue-Sp136 as TOSV genotype B; the isolates Fue-Sp40, Fue-Sp42, and Leg-Sp49 as ARBV. The sequences of the Fue-Sp149 isolate showed the highest similarity with the sequences relative to virus named Arrabida recently characterized in Portugal (Genebank; KC773871-3 and KF286394) [42]. For this reason Fue-Sp149 was tentatively named Arrabida-like virus.

**Table 2:** Virus isolation from *Phlebotomus perniciosus* in Fuenlabrada and Leganés collection sites

Virus isolated	Collection site code	Collection date	Sex	No. of sanflies	No. of pools	No. positive/total tested (MFIR)	Isolate name and GenBank acc. no.
TOSV	FUE-JIC	Aug-2012	M	23	1	1/366 (0.27)	Fue-Sp45 [EMBL:LN848240] [EMBL:LN848246] [EMBL:LN848251]
	FUE-BOS	Aug-2013	M	23	1	1/597 (0.18)	Fue-Sp136 [EMBL:LN848241] [EMBL:LN848247] [EMBL:LN848252]
TOTAL						2/963 (0.21)	
ARBV	FUE-BOS; LEG-POL	Aug-2012	M	40	2	3/366 (0.82)	Fue-Sp40 [EMBL:LN848245] [EMBL:LN848253] Leg-Sp49 [EMBL:LN848243] [EMBL:LN848249] [EMBL:LN848255]
	FUE-JIC	Jul-2012	F	24	1		Fue-Sp42 [EMBL:LN848242] [EMBL:LN848248] [EMBL:LN848254]
Arrabida virus	FUE-JIC	Jul-2013	M	21	1	1/597 (0.18)	Fue-Sp149 [EMBL:LN848244] [EMBL:LN848250] [EMBL:LN848256]

MFIR minimun field infection rate/100 sand flies, TOSV Toscana virus, ARBV Arbia virus, FUE Fuenlabrada, LEG Leganés, M male, *F* female

TOSV lineage B isolates were found in male sand fly pools trapped in the FUE-JIC site in August 2012 and in the FUE-BOS site in August 2013, demonstrating the focus stability for this virus. Both TOSV sequences were obtained from male sand fly pools. One ARBV isolate was obtained from a female pool collected in the FUE-JIC site in July 2012. Two isolates of this virus were also found in males collected in August 2012 and trapped from the stations located in FUE-BOS and LEG-POL. The Arrabida-like virus was isolated from a male pool collected in the FUE-JIC station in July 2013.

The overall Minimum Field Infection Rate (MFIR) was 0.27 and 0.18 for TOSV in 2012 and 2013, respectively, and 0.82 for ARBV and 0.18 for Arrabida-like virus.

### Serological relationships analysis

The viral isolates, molecularly characterized as TOSV, ARBV and Arrabida-like viruses, were assayed by PRN test. The results of the serological relationships with other selected Phleboviruses are reported in Table 3. The Spanish strains Fue-Sp45 and Fue-Sp42 were clearly neutralized by MIAFs against Italian TOSV (PRN_80_ = 40) and ARBV (20 < PRN_80_ < 40), respectively. No serological reactions were produced using MIAFs against viruses belonging to different serocomplexes. The Arrabida-like strain showed a low positivity (PRN_50_ = 1:10) only with MIAFs against viruses belonging to SFNV serocomplex, as TOSV and SFNV, while no serological relationship was detected with MIAFs against viruses belonging to other serocomplexes. On the basis of PRN test results, the Arrabida-like virus seems to be strongly correlated to but clearly distinct from other tested viruses belonging to the SFNV complex.

**Table 3:** Serological identification by Plaque Reduction Neutralization (PRN) test

	Fue-Sp45		Fue-Sp42		Fue-Sp149	
MIAFs	PRN_80_^a^	PRN_50_^b^	PRN_80_	PRN_50_	PRN_80_	PRN_50_
TOSV	40	40/80	0	0	0	10
SFNV	10/20	20	0	0	0	10
TEHRV	0	10/20	0	0	0	0
SFSV	0	0	0	0	0	0
ARBV	0	0	20/40	40/80	0	0
SALV	0	0	10	10/20	0	0

MIAF mouse immune ascitic fluid, PFU plaque forming units, PRN plaque reduction neutralization, TOSV Toscana virus, SFNV sand fly fever Naples virus, TEHRV Teheran virus, SFSV sand fly fever Sicilian virus, ARBV Arbia virus, SALV Salehabad virus

0 = <1:10; ^a^: values indicate highest MIAF dilution producing an PFU inhibition ≥80 %; ^b^: values indicate highest MIAF dilution producing an PFU inhibition ≥ 50 %

### Phylogenetic studies and comparative analysis

**Fig. 2:**
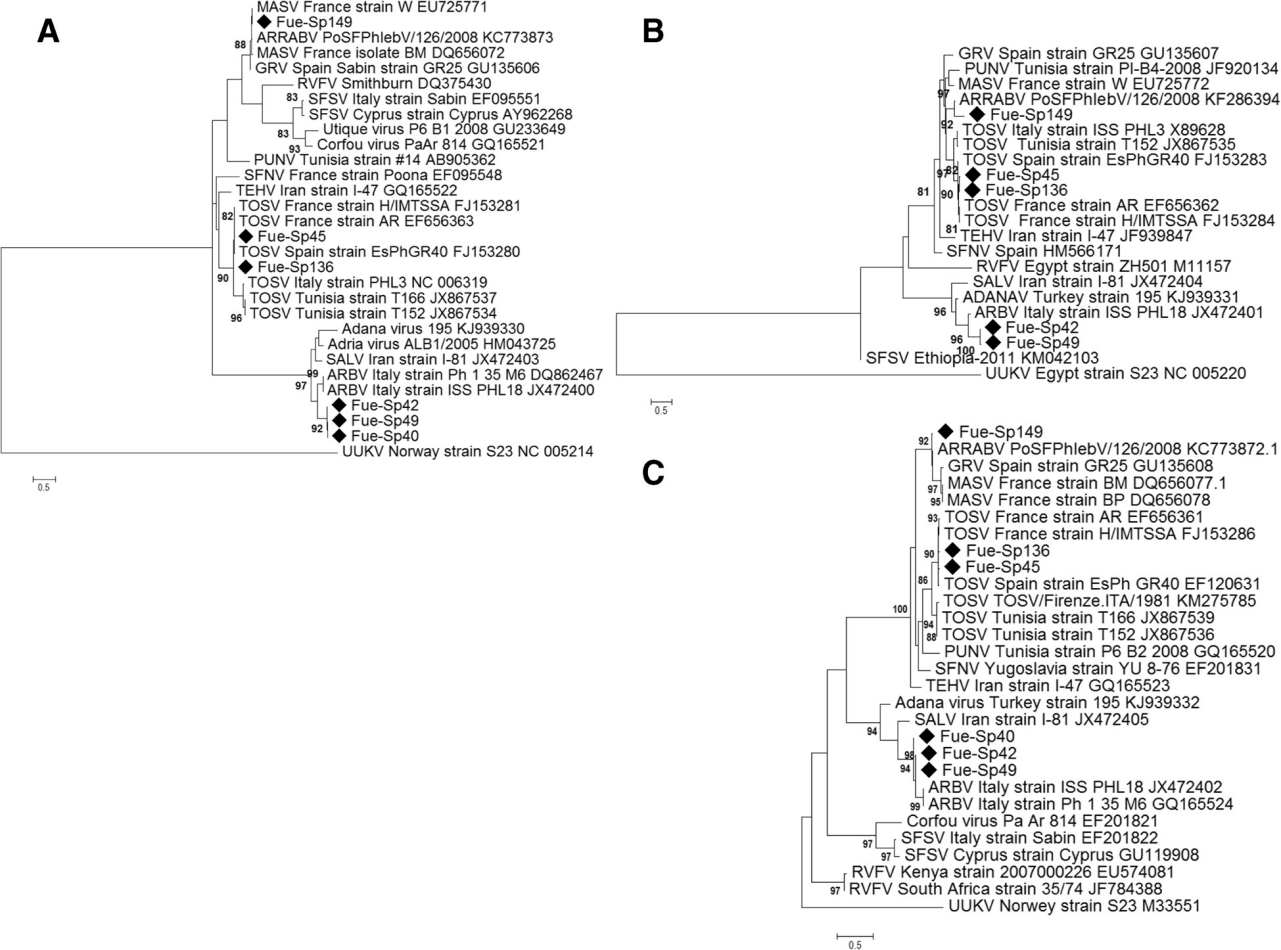
Phylogenetic trees of partial L, M and S segments of the phleboviruses isolated in Fuenlabrada and Leganés Municipalities. The phylogenetic trees were constructed using Kimura-2 parameter/Tamura 3 model for L (**a**) and M (**b**) segments and Maximum Parsimony model for S segment (**c**). The neighbour-joining method was used. Sequences information corresponds to Virus/Country isolation/Strain/GenBank Acc. Number. The sequences of the viral isolates are indicated with a black square. The bar indicates the percentage of diversity. Bootstrap values over 80 % obtained from 1000 replicate trees are shown for key nodes. MASV: Massilia Virus; GRV: Granada Virus; PUNV: Punique Virus; TOSV: Toscana Virus; TEHV: Teheran Virus; SFNV: Sand fly Fever Naples Virus; SFSV: Sand fly Fever Sicilian Virus; ARBV: Arbia Virus; SALV: Salehabad Virus; UUKV: Uukuniemi Virus

Nucleotide and amino acid analysis of the six viral isolates were performed on all three partial sequences of the L, M and S segments. From the analysis of the phylogenetic trees of the three segments analyzed, Fue-Sp45, Fue-Sp136 and Fue-Sp149 were shown to belong to the SFNV complex as well as Fue-Sp40, Fue-Sp42 and Leg-Sp49 were related to the SALV complex [Fig. 2a, b, c]. The relationships between the viruses of the SFNV complex were confirmed by the analysis of the genetic distances (Tables 4 and 5): at the nucleotide level, the distances between these isolates and viruses in the SFNV complex were within a range of 2–27 %, 4–29 % and 1–35 % for N, M and L proteins, respectively. The Fue-Sp45 and Fue-Sp136 clustered in the TOSV subgroup. The genetic distances for all three sequences analyzed showed that they were closely related to each other and to TOSV genotype B sequences (nucleotide composition homology ranging from 99 to 95 %) known to circulate in Spain and in France, confirming the presence of this TOSV lineage also in the populations of *P. perniciosus* in Madrid.

**Table 4:** Intergroup nucleotide diversity (%) of partial sequences of S (N gene), M (G2 gene) and L (L gene) segments of Fue- and Leg-Sp isolates and other Phleboviruses

	SFNV complex	SALV complex	SFSV	Courfou virus	Riftvalley virus	UUKV virus
Fue-Sp45 Fue-Sp136						
N	3–27	41–42	45–46	41	41–44	56
G2	4–27	44–47	44	–	44	58
L	1–35	38–41	36–39	37	40	50
Fue-Sp42 Fue-Sp40 Leg-Sp49						
N	37–41	10–27	45	37	40	43
G2	46–52	21–35	51	–	50	64
L	37–45	20–27	37	44	41	51
Fue-Sp149						
N	2–27	37–43	40–41	43	42	49
G2	17–29	45–52	43	–	45	62
L	2–33	41–47	36	35	32	52

SFNV Sand fly Fever Naples virus, SALV Salehabad virus, SFSV Sand fly Fever Sicilian virus, UUKV Uukuniemi virus

**Table 5:** Intragroup nucleotide diversity (%) of partial sequences of S (N gene), M (G2 gene) and L (L gene) segments of Fue- and Leg-Sp isolates and other Phleboviruses belonging to the same serogroup

	Fue-Sp136	TOSV Genotype A	TOSV Genotype B	SFNV	MASV	TEHV
Fue-Sp45						
N	3	14–15	3–4	22	27	25
G2	2	15–17	3–5	25–26	24	26
L	2	17–19	1–3	32–33	32–34	25
	Fue-Sp40	Leg-Sp49	ARBV	ADANAV	ADRIAV	SALV
Fue-Sp42						
N	3	0	9	28	–	22
G2	–	1	21	27–28	–	35–36
L	0	1	20	26–27	27	23–24
	MASV	GRNV	Arrabida virus	PUNV	TOSV	TEHV
Fue-Sp149						
N	12–13	13	2	19	24–27	24
G2	25	26	17	28	26–28	29
L	2–4	3	3	30	32–34	31

TOSV Toscana virus, SFNV Sand fly Fever Naples virus, MASV Massilia virus, TEHV Teheran virus, ARBV Arbia virus, SALV Salehabad virus, GRNV Granada virus, PUNV Punique virus

The relatedness of Fue-Sp40, Fue-Sp42 and Leg-Sp49 isolates with viruses belonging to the SALV complex, in accordance with the serological assay, was demonstrated from nucleotide distance analysis (10–27 %, 21–35 % and 20–27 % in nucleotide composition for N, G2 and L proteins, respectively) (Tables 4 and 5). The analysis of all three sequences of these isolates was distantly related to the other *Phlebovirus* groups (*P* distance ≥ 37 % in nucleotide composition). Analysis of the variation of nucleotide identities within members of the SALV complex indicated that these viruses were more closely related to the Italian ARBV sequences, with a similarity of 80, 79 and 91 % on L, G2 and N nucleotide sequences, respectively.

As shown in Table 5, the analysis of the G2 and N partial sequences of Fue-Sp149 showed that this virus was closely related to but clearly distinct from the groups of MASV and GRV sequences. Indeed, while the *p*-distance values of Fue-Sp149 L partial sequence with MASV and GRV were 2–4 % and 3 %, respectively, G2 partial sequences was 25 and 26 %, respectively. In addition, N sequences diversity between this isolate and MASV and GRV was 12–13 % and 13 %, respectively. Fue-Sp149 L and N nucleotide sequences resulted closely related in nucleotide composition (97 and 98 %, respectively) to Arrabida virus sequences recently described in Portugal [42]. The G2 partial sequence of Fue-Sp149 showed a 17 % of diversity with G2 sequence of Arrabida virus from Portugal. All these results were confirmed by the analysis of the amino acid translation (data not shown).

## Discussion

Active entomological and virological surveillance is an important approach to provide early warning and predictive capacity about the risk of the *Ph*B-pathogens epidemics. The present study was carried out in the context of the EU FP7 EDENext project (http://www.edenext.eu) with the aim to monitor the *Ph*B-viruses presence in sand flies collected in Fuenlabrada and Leganés Municipalities, areas of Madrid known for a recent outbreak of human cutaneous and visceral leishmaniasis and where the presence of *L. infantum* in *P. perniciosus* has been also demonstrated [21, 30]. From our investigation, six viral isolates have been obtained from *P. perniciosus* pools collected in the same area and serological and molecular characterization showed that these Phleboviruses were belonging to two different serocomplexes of the *Phlebovirus* genus.

Infections by *Phlebovirus* and *Leishmania* represent today an important public health problem in countries in which these microorganisms circulate [43]. Indeed, these pathogens are known to be transmitted by the same sand fly vector, and the close relationships between human leishmaniasis and phleboviral infections are now widely reported [3, 22]. Indeed, the co-circulation of *L. infantum* and TOSV in the sand fly vector *P. tobbi* and *L. tropica* and TOSV in the sand fly vector *P. sergenti* were reported in Cyprus [24] and Morocco [25], respectively. In addition, even if no dual infections were observed, *L. infantum* and TOSV and MASV were detected in *P. perniciosus* collected from the same trapping site in Marseille urban area [23].

In the ecological spread of *Ph*B-pathogens, the presence of insect vectors is an important prerequisite for transmission; however, it is not the only factor determining whether or not a pathogen can be established. In particular local environmental components (i.e. climate, host seeking, presence vertebrate reservoir, accessibility to humans) represent factors influencing the pathogens amplification and the diffusion of the diseases transmitted by them. Interestingly, in this periurban area of Madrid, following afforestation started in 2005 and finished in 2011 [31], a large population of hares, very close to the urban settlements, was observed where an outbreak of leishmaniasis occurred. The environmental changes caused by humans have probably modified the ecology of these leporids, moving from a woodland cycle to an urban one [31]. The study of blood meal preferences of *P. perniciosus* females [30] indicated that these lagomorphs are frequently bitten by *P. perniciosus* sand flies, strongly suggesting they contribute to the maintenance of high sand fly populations in this epidemic area [29, 30, 44]. Recent studies provided significant information about the role of Iberian hares as sylvatic reservoirs of *Leishmania* highlighting the infectivity of apparently healthy *L. infantum* infected hares (*L. granatensis*) to phlebotomine sand flies (*P. perniciosus*) [31].

At present, limited informations are available about Phleboviruses seroprevalence both in lagomorphs and in other vertebrates. However all the above-mentioned evidences could suggest that the presence of this unexpected ecological niche of Phleboviruses in Madrid foci could be the result of the massive presence of the vertebrates potential reservoirs, (e.g. rabbits and hares) and *P. perniciosus*. However the role of vertebrates in the maintenance of the *Ph*B-viruses transmission cycle remains unclear [1]. To date, neither mammals nor birds have been recognized as potential reservoirs, although few studies have been carried out on these vertebrates. Verani et al. reported a single isolation of TOSV strain, in Italy, from the brain of the bat *Pipistrellus kuhli* which was trapped in areas where *P. perniciosus* and *P. perfiliewi* were present [38, 45]. Recently seroprevalence studies on dogs in Turkey strongly suggested that canine species could be a possible candidate reservoir of TOSV [26].

The detection of several isolates belonging to different *Phlebovirus* serocomplexes from *P. perniciosus* confirmed the important role of this sand fly species as vector, being the main natural vector of the other Phleboviruses, such as GRV and TOSV, previously identified in Spain [15, 19]. The presence of TOSV and ARBV from males of sand fly pools (Table 2) clearly pointed out that the venereal or vertical transmission may be a successful amplification mechanism of these viruses in nature, in agreement with previous experimental infections studies [36, 46, 47]. Arrabida-like virus was also found in a male pool of sand flies suggesting that also for this virus the amplification in nature by venereal or vertical transmission is possible. Fue-Sp149 isolate was 98, and 93 % identical, at nucleotide level of N and L genes respectively, to the recently characterized *Phlebovirus* Arrabida, belonging to SFNV complex, isolated by Phlebotomine sand flies in South Portugal. The recent published data on genome sequencing suggested that this Arrabida virus from Portugal could be considered a reassortant of GRV and MASV, donors of the long and short segments, with an unknown *Phlebovirus*, donor of the medium segment [42]. According to findings of Amaro et al. [42], Fue-Sp149 showed close relationships with MASV and GRV with a low nucleotide divergence on N and L partial sequences (Table 5). Furthermore, the calculated *P* distance in M segment G2 gene between MASV, GRV and Arrabida virus and Fue-Sp149 was 25, 26 and 17 % at nucleotide level, respectively. The differences in the M segment are a common distinctive characteristic among *Phlebovirus* and is frequently the basis for the distinction among different Phleboviruses. Indeed GRV is a natural reassortant of MASV, donor of the long and short segments, with a yet unidentified *Phlebovirus,* donor of the medium segment. The full FueSp-149 isolate genome characterization (in particular the M segment) will clarify the possible presence of a new reassortant *Phlebovirus* genetically related to Arrabida virus. Pending the results of the full genome characterization, the FueSp-149 isolate has been considered as putative novel virus tentatively named Arrabida-like virus. Due to the Arrabida-like virus, which belongs to the SFNV complex and includes many viruses able to infect humans (e.g., TOSV, SFNV, GRV, MASV) [1], the impact on public health in the studied area should be investigated.

In the present study, for the first time, the ARBV presence was demonstrated in Spain. The analysis of the phylogenetic trees showed some differences between the Spanish and Italian ARBVs (Fig. 2). Indeed, the preliminary analysis of the partial sequences of S, M and L segments showed differences between each position of the nucleotide triplet coding for the amino acid sequences (data not shown). Further studies will be necessary to determine if ARBV from Spain might represent a new geographical lineage inside ARBV serotype.

The isolation of TOSV lineage B confirmed the stability of the natural focus active in Madrid community. Even if no recent epidemiological data on human population in the area of study are reported, seroprevalence analysis confirmed the TOSV circulation in human population in Spain and in Madrid area [48, 49]. More recently the epidemiological bulletin of Madrid (Boletín Epidemiológicode la Comunidad de Madrid N° 10, volumen 20 Octubre 2014) reported several cases of viral meningitis of unknown etiology (67 %) mainly in the urban area close to the green park investigated (Alcorcón, Leganés and Fuenlabrada). In the context of public health, the isolation of TOSV lineage B in these areas should be taken into account. Indeed, all these data suggest a possible role of TOSV as responsible of meningitis cases being, up to now, the only neurotropic *Phlebovirus* known to be circulating in Madrid region. In addition the MFIR of 0.21 % for TOSV, obtained from sand flies collected in Fuenlabrada in 2012–2013 summers, was higher than the infection rate of 0.05 % up to now found in Spain [15] but similar to that reported in Italy (MFIR = 0.2 %) [38] where yearly TOSV meningitis cases are known to occur in the summer [6, 49–51]. For this reason TOSV should be included in routine laboratory diagnosis of diseases with neurological symptoms occurring in south-west Madrid area.

## Conclusion

The present study demonstrated the presence of different *Phlebovirus* species in a recently identified human leishmaniasis focus in south-west of Madrid area showing the co-circulation of both pathogens. Based on our findings, an active field-based study, that combines entomological, parasitological and virological aspects, results to be a valid approach to monitor the possible appearance of vector-borne emerging diseases. A better knowledge of *Ph*B-viruses epidemiological aspects may be important in public health to prevent epidemics and/or spreading of these viruses in geographic areas where no evidence of their circulation has been highlighted.

## Abbreviations

ARBV: Arbia virus
CPE: cytopathic effect
F: female
FUE: Fuenlabrada
GRNV: Granada virus
*L*: *Leishmania*
LEG: Leganés
M: male
MASV: Massilia virus
MFIR: Minimun Field Infection Rate/100 sand flies
MIAF: mouse immune ascitic fluid
*P*: *Phlebotomus*
PFU/mL: Plaque Forming Units/mL
*Ph*B: *Phlebotomus*-borne
PRN: plaque reduction neutralization
PUNV: Punique Virus
RT-PCR: Reverse Transcriptase (RT)-PCR
SALV: Salehabad Virus
SFNV: Sandfly Fever Naples Virus
SFSV: Sand fly Fever Sicilian Virus
TEHV: Teheran Virus
TOSV: Toscana Virus
UUKV: Uukuniemi Virus
